# Impact of preceding respiratory viral infections on the clinical severity of patients with pneumococcal pneumonia

**DOI:** 10.1111/irv.12265

**Published:** 2014-06-24

**Authors:** Young Kyung Yoon, Kyung Sook Yang, Jang Wook Sohn, Chang Kyu Lee, Min Ja Kim

**Affiliations:** a Division of Infectious Diseases, Department of Internal Medicine, Korea University College of MedicineSeoul, Korea; b Institute of Emerging Infectious Diseases, Korea University College of MedicineSeoul, Korea; c Department of Biostatistics, Korea University College of MedicineSeoul, Korea; d Department of Laboratory Medicine, Korea University College of MedicineSeoul, Korea

**Keywords:** Clinical severity, pneumonia, respiratory viruses, *Streptococcus pneumoniae*

## Abstract

**Background:**

This study aimed to investigate the impact of preceding respiratory viral infections (RVI) on the clinical severity of pneumococcal pneumonia patients.

**Methods:**

A retrospective observational study was conducted at a university hospital from January 2009 to March 2013. Study subjects included adults (aged ≥18 years) with pneumococcal pneumonia who had undergone laboratory tests for RVI. Multivariate logistic regression analysis was performed to identify risk factors associated with severe pneumococcal pneumonia, defined as severity with the Pneumonia Severity Index (PSI) score ≥91.

**Results:**

In total, 191 patients with pneumococcal pneumonia were included for analysis and stratified into 2 groups: the severe group with a PSI score ≥91 (*n* = 99) and the non-severe group with a PSI score <91 (*n* = 92). Preceding RVIs were detected in 48 patients, including influenza A virus (*n* = 20), influenza B virus (*n* = 4), parainfluenza viruses (*n* = 5), metapneumovirus (*n* = 4), rhinovirus (*n* = 4), respiratory syncytial viruses (*n* = 6), coronaviruses (*n* = 2), and mixed viral infections (*n* = 3). In the multivariate logistic regression analysis, preceding RVIs (odds ratio [OR], 2·49; 95% confidence interval [CI], 1·10–5·60), male sex (OR, 2·58; 95% CI, 1·24–5·38), old age (OR, 2·92; 95% CI, 1·37–6·24), hypoalbuminemia (OR, 3·26; 95% CI, 1·56–6·84)], and azotemia (OR, 2·24; 95% CI, 1·08–4·67) were significantly associated with severe pneumococcal pneumonia.

**Conclusion:**

This study suggests that preceding RVIs might be one of the risk factors affecting the clinical severity of pneumococcal pneumonia.

## Background

Community-acquired pneumonia (CAP) is the leading cause of death due to infectious diseases worldwide and accounts for major morbidity, mortality, and cost of care.^1^,^2^ *Streptococcus pneumoniae* is the most dominant bacterial cause of CAP in adults.^3^ Despite advances in medical care, mortality from pneumococcal pneumonia still ranges from 11% to 20%.^4^,^5^ Known prognostic factors for mortality due to pneumococcal pneumonia include old age, male sex, pre-existing lung diseases, solid organ tumors, nosocomial infections, leukopenia, low body temperature, urea nitrogen level >30 mg/dl, hypoalbuminemia, hypoxemia, septic shock, and high severity scores.^6^–^9^ However, preceding respiratory virus infection (RVI) as a potential risk factor for severe pneumococcal pneumonia was not evaluated in these studies due to the unavailability of routine virological diagnostic assays.^6^–^9^ It has been observed that polymerase chain reaction (PCR)-based testing allows the detection of various respiratory viruses and viral–bacterial co-infection might be associated with severe diseases.^10^–^14^

Rapid molecular diagnostic techniques, such as multiplexed nucleic acid PCR assays for respiratory viral pathogens, have recently been introduced into clinical practice. In recent studies, one or more respiratory virus infections have been reported in hospitalized adult patients with CAP.^10^–^12^ Particularly, preceding RVIs have long been regarded as a predisposing factor for pneumococcal pneumonia.^12^,^15^–^18^ Viral infections cause changes in respiratory tracts, including bronchoconstriction, increased mucus production,^19^ stronger adhesion of pneumococci to virus-infected cells than uninfected cells,^20^ decreased ciliary action,^21^ damage to mucosal cells, and dysfunction of leukocytes.^20^ However, studies regarding the clinical impact of preceding RVIs in patients with pneumococcal pneumonia are limited.

The purpose of this study was to evaluate the clinical significance of preceding RVIs on the clinical severity of pneumococcal pneumonia in adults.

## Methods

### Study design

This was a retrospective case–control study, which was performed at a 950-bed tertiary care hospital in Seoul, the Republic of Korea, from January 2009 to March 2013. Subjects included adult patients (age ≥18 years) with pneumococcal pneumonia who had been tested for RVI using multiplex reverse transcription-polymerase chain reaction (RT-PCR) within 30 days preceding *S. pneumoniae* isolation and were followed up until death or hospital discharge. A case was defined as a patient with severe pneumonia (i.e., determined by a pneumonia severity index [PSI] score ≥91 [risk class ≥IV]), while a control was defined as an adult patient with non-severe pneumonia (i.e., PSI score ≤90 [risk class ≤III]).^22^

The study protocol was approved by the hospital institutional review board, which also waived the requirement of informed consent, as this retrospective study required no deviation from routine medical practice.

### Definitions and data collection

Pneumococcal pneumonia was defined as an acute lower respiratory tract infection with opacity or infiltrates on a chest radiograph as confirmed by radiologists plus isolation of *S. pneumoniae* from sputum samples in outpatients or inpatients within 48 hours of hospital admission. All patients received antimicrobial therapy for >5 days. Acute lower respiratory tract infection was defined as the presence of two or more of the following symptoms or signs: productive cough, fever, dyspnea, pleuritic chest pain, and crackles. Septic shock was defined according to standard criteria.^23^

Clinical data for each patient diagnosed with pneumococcal pneumonia were collected from a computerized hospital database. Only a single episode per patient was included in this study during all winter seasons. Patients who did not undergo laboratory tests for RVI within 30 days preceding pneumococcal pneumonia were excluded. Hospital-acquired pneumonia cases that were presented ≥48 hours after admission or within 7 days after hospital discharge were also excluded. All patients were treated with antibiotics according to local practice guidelines.^24^

Clinical parameters for analysis included demographic and clinical characteristics, comorbid medical conditions,^25^ pneumonia treatment, antimicrobial susceptibility, and treatment outcome. The PSI^22^ and CURB-65^26^ scores were calculated based on the clinical presentation of pneumonia. Pneumonia-related mortality was defined according to microbiological failure, persistent pneumonia-associated symptoms and signs, and the absence of other definite causes of death.

### Microbiological methods

Blood and sputum cultures were routinely performed for patients who were present at the emergency room or were admitted to the hospital due to suspected pneumonia. Simultaneously, patients who had preceding or concurrent flu-like or cold symptoms underwent nasopharyngeal swab sampling to isolate the respiratory viruses. Clinical isolates of *S. pneumoniae* were identified by conventional biochemical methods and the VITEK 2 GP card (bioMérieux, Marcy l'Etoile, France). Antimicrobial susceptibility was determined using the VITEK 2 system according to the revised Clinical and Laboratory Standards Institute's interpretive criteria for *S. pneumoniae*.^27^

Nasopharyngeal swab samples were tested for 12 respiratory virus pathogens, including influenza virus type A and type B, human metapneumovirus (HMPV), respiratory syncytial virus (RSV) type A and type B, rhinovirus, parainfluenza viruses (PIV; types 1, 2, and 3), coronaviruses OC43/229E and NL63, and adenovirus, by using the Seeplex RV assay (Seegene, Inc., Seoul, Korea) based on a multiplex RT-PCR method. Viral DNA and RNA were extracted from each respiratory specimen using the Gene-spin™ kit (iNtRON Biotechnology, Seoul, Korea) according to the manufacturer's instructions and the nucleic acid amplification was conducted using the Seeplex RV master mix as described previously.^28^

### Statistical analyses

Demographic and clinical characteristics were compared between patients with severe and non-severe pneumococcal pneumonia. Independent categorical variables were described using counts (proportions) and compared using the chi-square test or Fisher's exact test. Continuous variables were expressed as mean ± standard deviation or median interquartile range (IQR). A two-sample Student's *t*-test was used to compare continuous independent variables with normal distribution. A Mann–Whitney *U*-test was used to compare continuous independent variables with a non-normal distribution. Multivariate logistic regression analyses using a backward stepwise variable selection based on logistic regression statistics was used to examine the impact of multiple independent predictors on the clinical severity of pneumococcal pneumonia as a dependent variable. Hosmer–Lemeshow goodness-of-fit tests were performed to evaluate the models. Internal accuracy obtained by leave-one-out cross-validation was used to evaluate the performance of the predictive model. Statistical significance was defined as a *P* < 0·05. Statistical analyses were performed using ibm spss Statistics version 20.0 (IBM Corporation, Armonk, NY, USA), r 2.15.2 (The R Foundation for Statistical Computing, Vienna, Austria), and sas 9.2 (SAS Institute Inc., Cary, NC, USA).

## Results

### Patients and clinical characteristics

During the study period, a total of 504 694 patients visited our hospital as outpatients (*n* = 410 856) or inpatients (*n* = 93 838). A total of 975 patients (1003 multiple episodes) who had *S. pneumoniae* isolation from sputum cultures were initially screened. Of these patients, 900 were aged ≥18 years. Patients who did not undergo multiplex RT-PCR for RVI within 30 days preceding *S. pneumoniae* isolation (*n* = 640), received antibiotic therapy for <5 days (*n* = 51), and acquired pneumonia at the hospital (*n* = 18) were excluded from the study. Finally, 191 patients who had pneumococcal pneumonia were analyzed for the study, including 26 (13·6%) outpatients and 165 (86·4%) hospitalized patients. None of them had polymicrobial infection or any simultaneous infections at other sites.

The median (IQR) of the CURB-65 and PSI scores in the 191 patients with pneumococcal pneumonia were 2 (1–4) and 92 (74–117), respectively. PSI classes were observed as follows: class II (*n* = 38, 19·9%), class III (*n* = 53, 27·7%), class IV (*n* = 71, 37·2%), and class V (*n* = 29, 15·2%). Ninety-nine (51·8%) patients had severe pneumococcal pneumonia (PSI score ≥91).

**Figure 1:**
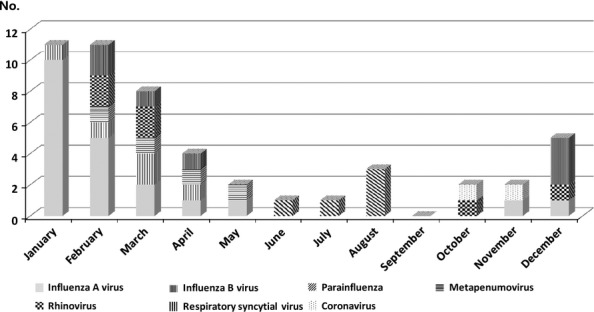
The monthly distribution of the etiology of preceding or concurrent respiratory viral infections in patients with pneumococcal pneumonia from January 2009 to March 2013.

One hundred and thirty-two (66·3%) patients received RVI testing throughout the influenza seasons of the Northern hemisphere (November to April). Forty-eight (25·1%) patients had preceding or concurrent RVIs detected by multiplexed RT-PCR performed within 30 days of pneumonia presentation (Table1). In particular, Influenza A and B and RSV infections were predominant during the winter months of the study period (January 2009 to March 2013) as shown in Figure1.

**Table 1:** The etiology of respiratory viral infections in patients with pneumococcal pneumonia

Type of virus	*n* (%)
Influenza A virus	20 (41·7)
Influenza B virus	4 (8·3)
Parainfluenza viruses	5 (10·4)
Parainfluenza virus 1	2 (4·2)
Parainfluenza virus 3	1 (2·1)
Parainfluenza virus 4	2 (4·2)
Human metapneumovirus	4 (8·3)
Rhinovirus	4 (8·3)
Respiratory syncytial viruses	6 (12·5)
Respiratory syncytial virus A	3 (6·3)
Respiratory syncytial virus B	3 (6·3)
Coronaviruses	2 (5·4)
Coronavirus 229E/NL63	2 (5·4)
Mixed viruses*	3 (6·3)
Total	48 (100)

* Mixed viruses represent co-infection of two viruses: Respiratory syncytial virus A plus influenza A virus (*n* = 1), metapneumovirus plus influenza B (*n* = 1), and rhinovirus plus coronavirus 229E/NL63 (*n* = 1).

Comparisons of demographic and clinical characteristics between the patients with severe and non-severe pneumococcal pneumonia are shown in Table2. Hypoalbuminemia, azotemia, and thrombocytopenia were more common in the case group (severe pneumococcal pneumonia) than in the control group (non-severe pneumococcal pneumonia). In addition, anemia and hyperbilirubinemia were more common in the case group (Table2). The case group had more frequent preceding RVIs than the control group.

**Table 2:** Demographic and clinical characteristics of 191 patients with pneumococcal pneumonia according to clinical severity

Variables	All (*n* = 191)	Severe group (*n* = 99, 51·8%)	Non-severe group (*n* = 92, 48·2%)	*P*-value
Male sex, *n* (%)	119 (62·3)	76 (76·8)	43 (46·7)	<0·001
Age (year), median (IQR)	70 (60–77)	73 (67–79)	64 (54–74)	<0·001
Age ≥65 years, *n* (%)	123 (64·4)	78 (78·8)	45 (48·9)	<0·001
Preceding viral infection, *n* (%)	48 (25·1)	32 (32·3)	16 (17·4)	0·017
Interval between pneumonia and viral infection (day), median (IQR)	1 (1–3)	3 (0–8)	1 (0–7)	0·295
Comorbidity, *n* (%)				
Cardiovascular	33 (17·3)	17 (17·2)	16 (17·4)	0·968
Central nervous system	37 (19·4)	24 (24·2)	13 (14·1)	0·077
Malignancy	34 (17·8)	25 (25·3)	9 (9·8)	0·005
Diabetes mellitus	44 (23·0)	32 (32·3)	12 (13·0)	0·002
Renal	7 (3·7)	3 (3·0)	4 (4·3)	0·713
Hepatic	6 (3·1)	3 (3·0)	3 (3·3)	1·000
Respiratory	36 (18·8)	19 (19·2)	17 (18·5)	0·900
Hematological	9 (4·7)	7 (7·1)	2 (2·2)	0·172
Connective tissue diseases	3 (1·6)	0	3 (3·3)	0·110
Charlson comorbidity score*	2 (0–4)	3 (1–5)	1 (0–3)	<0·001
Pneumococcal bacteremia, *n* (%)	11 (5·8)	9 (9·1)	2 (2·2)	0·040
Predisposing factors, *n* (%)				
Prior operation	13 (6·8)	8 (8·1)	5 (5·4)	0·468
Receipt of corticosteroids	21 (11·0)	10 (10·1)	11 (12·0)	0·682
Laboratory results				
White blood cell count ≥15 000/μl, *n* (%)	65 (34·0)	42 (42·4)	23 (25·0)	0·011
C-reactive protein >100 mg/dl, *n* (%)	113 (59·2)	67 (67·7)	46 (50·0)	0·013
Procalcitonin ≥2 ng/ml, *n* (%)	29 (15·2)	22 (22·2)	7 (7·6)	0·005
Hematocrit <30%, *n* (%)	61 (31·9)	42 (42·4)	19 (20·7)	0·001
Platelet count <100 000/μl, *n* (%)	31 (16·2)	23 (23·2)	8 (8·7)	0·006
Albumin <3·0 mg/dl, *n* (%)	79 (41·4)	58 (58·6)	21 (22·8)	<0·001
Bilirubin ≥2 mg/dl, *n* (%)	20 (10·5)	15 (15·2)	5 (5·4)	0·028
Blood urea nitrogen ≥19 mg/dl, *n* (%)	96 (50·3)	67 (67·7)	29 (31·5)	<0·001

IQR, interquartile range; PSI, pneumonia severity index.

* Charlson comorbidity score, CURB-65, and pneumonia severity index scores were assessed on the day of community-acquired pneumonia diagnosis.

The case group had more multilobar consolidation on the chest radiographs than the control group (32 [32·3%] versus 15 [16·3], respectively; *P* = 0·010). However, there was no significant difference in the complicated pleural effusion between the case and control groups (8 [8·1%] versus 4 [4·3], respectively; *P* = 0·288).

### Antimicrobial treatment and clinical outcome

The treatment and clinical outcomes between the severe and non-severe groups are compared in Table3. There were no significant differences in antimicrobial therapy, intensive care units, hemodialysis, and mechanical ventilation between the two groups. All patients from the two groups received the appropriate antibiotic therapy for pneumococcal pneumonia based on the antimicrobial susceptibility results. Twenty-three (12·0%) patients received antibiotics for pneumococcal pneumonia prior to arriving at this hospital, which was not significantly different between the case and the control groups (14 [14·1%] vs. 9 [9·8%], respectively; *P* = 0·355). The resistance rates of the pneumococcal isolates to penicillin and levofloxacin were significantly higher in the case group than in the control group (Table4).

**Table 3:** Antimicrobial treatment and clinical outcome in 191 patients with pneumococcal pneumonia according to clinical severity

Variables	All (*n* = 191)	Severe group (*n* = 99, 51·8%)	Non-severe group (*n* = 92, 48·2%)	*P* value
Antiviral treatment (Oseltamivir), *n* (%)	17 (8·9)	11 (11·1)	6 (6·5)	0·266
Antibiotic treatment, *n* (%)				
Cephalosporins	124 (64·9)	70 (70·7)	54 (58·7)	0·082
β-lactam/β-lactamase inhibitors	4 (2·1)	1 (1·0)	3 (3·3)	0·353
Fluoroquinolones	91 (47·6)	48 (48·5)	43 (46·7)	0·809
Carbapenems	11 (5·8)	7 (7·1)	4 (4·3)	0·420
Glycopeptides	12 (6·3)	8 (8·1)	4 (4·3)	0·288
Macrolides	67 (35·1)	30 (30·3)	37 (40·2)	0·151
Piperacillin/tazobactam	23 (12·0)	15 (14·1)	8 (9·8)	0·355
Clindamycin	11 (5·8)	7 (7·1)	4 (4·3)	0·420
Other	7 (3·7)	6 (6·1)	1 (1·1)	0·120
Intensive care unit care	50 (26·2)	38 (38·4)	12 (13·0)	<0·001
Mechanical ventilator care	29 (15·2)	22 (22·2)	7 (7·6)	0·005
Hemodialysis	2 (1·0)	2 (2·0)	0	0·498
Clinical outcome				
Hospital stay (day)*, median (IQR)	8 (4–18)	8 (6–17)	6 (3–14)	0·006
Pneumococcal-related mortality, *n* (%)	12 (6·3%)	11 (11·1)	1 (1·1)	0·004
In-hospital mortality, *n* (%)	16 (8·4)	11 (11·1)	5 (5·4)	0·157

IQR, interquartile range.

* Inpatient hospital days.

**Table 4:** Antimicrobial resistance rates of *Streptococcus pneumoniae* isolates from 191 patients with pneumococcal pneumonia according to clinical severity

Variables	All (*n* = 191)	Severe group (*n* = 99, 51·8%)	Non-severe group (*n* = 92, 48·2%)	*P* value
Penicillin	13 (6·8)	12 (12·1)	1 (1·1)	0·002
Third generation cephalosporins	38 (19·9)	22 (22·2)	16 (17·4)	0·403
Erythromycin	122 (63·9)	64 (64·6)	58 (63·0)	0·818
Levofloxacin	15 (7·9)	12 (12·1)	3 (3·3)	0·023
Imipenem	64 (33·5)	32 (32·3)	32 (34·8)	0·719
TMP/SMX	105 (55·0)	53 (53·5)	52 (56·5)	0·679

TMP/SMX, trimethoprim/sulfamethoxazole.

The all-cause in-hospital mortality rate and pneumonia-related mortality rate were 8·4% and 6·3%, respectively. The median length of hospital stay for inpatients was 8 days (IQR, 4–18). Patients with severe pneumococcal pneumonia showed higher pneumonia-related mortality and longer hospital stays than patients with non-severe pneumococcal pneumonia (Table3).

### Impact of preceding RVIs on clinical severity

In the multivariate logistic regression analysis, preceding RVI (odds ratio [OR], 2·49; 95% confidence interval [CI], 1·10–5·60), male sex (OR, 2·58; 95% CI, 1·24–5·38), old age (OR, 2·92; 95% CI, 1·37–6·24), hypoalbuminemia (OR, 3·26; 95% CI, 1·56–6·84), and azotemia (OR, 2·24; 95% CI, 1·08–4·67) were significantly associated with severe pneumococcal pneumonia (Table5). The *P*-values for the Hosmer–Lemeshow goodness-of-fit test were >0·05 (*P* = 0·107). Hence, there was no significant evidence of a lack of fit for any of the final models.

**Table 5:** Multivariable logistic regression analysis of risk factors associated with severe pneumococcal pneumonia in 191 patients with pneumococcal pneumonia*

Variables	Odds ratio	95% confidence interval	*P*-value
Preceding respiratory viral infection	2·49	1·10–5·60	0·028
Male sex	2·58	1·24–5·38	0·012
Age ≥65 years	2·92	1·37–6·24	0·006
Albumin < 3·0 mg/dl	3·26	1·56–6·84	0·002
Blood urea nitrogen ≥19 mg/dl	2·24	1·08–4·67	0·031
Underlying diabetes mellitus	2·12	0·87–5·17	0·098
Underlying malignancy	2·36	0·91–6·12	0·079

* This model includes risk factors, such as male sex, old age (≥65 years), underlying diabetes mellitus, underlying malignancy, Charlson comorbidity score, preceding respiratory virus infection, septic shock, CURB-65 score, multilobar consolidation on the chest radiograph, white blood cell count ≥15 000/μl, C-reactive protein >100 mg/dl, procalcitonin ≥2 ng/ml, hematocrit <30%, platelet count <100 000/μl, albumin <3·0 mg/dl, bilirubin ≥2 mg/dl, and blood urea nitrogen ≥19 mg/dl.

Leave-one-out cross-validation was performed to assess the predictive accuracy of the final model. The AUCs for the clinical severity model were >0·80 for both the raw data set and the leave-one-out cross-validation. For the leave-one-out cross-validation, the sensitivity, specificity, positive predictive value, and negative predictive value obtained with an optimal cut-off point were >0·70 (Table6).

**Table 6:** The predictive probability of high severity pneumococcal pneumonia in the multivariate logistic regression model and validation results

Validation	AUC (95% CI)	% (95% CI)			

		Sensitivity	Specificity	PPV	NPV
Raw data set	0·819 (0·757–0·871)	88·9 (81·0–94·3)	65·2 (54·6–74·9)	73·3 (64·5–81·0)	84·5 (74·0–91·5)
LOOCV	0·811 (0·748–0·864)	78·8 (69·4–86·4)	73·9 (63·7–82·5)	76·5 (67·0–84·3)	76·4 (66·2–84·8)

AUC, area under the curve; 95% CI, 95% confidence interval; LOOCV, leave-one-out cross-validation; NPV, negative predictive value; PPV, positive predictive value.

During the study period, 142 adult patients with other forms of bacterial pneumonia underwent multiplex RT-PCR tests for RVI within 30 days preceding other types of bacterial isolation. Out of them, 25 (16·6%) patients had co-infection with RVIs and other forms of bacterial pneumonia, such as *Staphylococcus aureus* (*n* = 14), *Haemophilus influenzae* (*n* = 2), *Pseudomonas aeruginosa* (*n* = 3), and *Klebsiella pneumoniae* (*n* = 6). Preceding RVIs were detected in 25 patients, including influenza A virus (*n* = 9), HMPV (*n* = 2), rhinovirus (*n* = 8), RSV (*n* = 3), and mixed viral infections (*n* = 3: RSV plus coronavirus, coronavirus plus PIV, and rhinovirus plus PIV). In patients with preceding RVIs and other forms of bacterial pneumonia, compared to those with post-viral pneumococcal pneumonia, the median (IQR) of CURB-65 (2 [2–3] versus 2 [1–4], respectively; *P* = 0·720), PSI scores (118 [86–150] versus 100 [81–127], respectively; *P* = 0·201), severe pneumococcal pneumonia (10 [40·0%] versus 10 [20·8%], respectively; *P* = 0·081), all-cause in-hospital mortality rate (4 [16·0%] versus 4 [8·3%], respectively; *P* = 0·320), and pneumonia-related mortality rate (5 [20·0%] versus 4 [8·3%], respectively; *P* = 0·150) were not significantly different. However, in 142 adult patients with other forms of bacterial pneumonia, preceding RVIs were not significantly associated with severe pneumonia (5/25 [20·0%] in patients with severe pneumonia versus 19/117 [16·2%] in patients with non-severe pneumonia; *P* = 0·649).

## Discussion

In this study, we investigated the impact of preceding RVIs on the clinical severity of pneumococcal pneumonia. Preceding RVIs, male sex, old age, hypoalbuminemia, and azotemia were identified as risk factors that were significantly associated with severe pneumococcal pneumonia (PSI score ≥91) through validation of a multivariate model designed to predict clinical severity.

In this study, at least one respiratory virus was detected in 25·1% (48/191) of patients within the 30 days preceding pneumonia presentation. The most common viral pathogens detected in decreasing frequency were influenza A virus, RSV, and PIV. According to three prospective studies, bacterial–viral co-infections were present in 4–30% of adults with CAP.^10^–^12^ In a study by Jennings *et al*.,^12^ rhinovirus was the most common virus identified from mixed viral and bacterial infections in adults with CAP, followed by RSV and influenza A virus. Our findings indicate that a quarter of patients presenting pneumococcal pneumonia had RVIs in the preceding 30 days.

In this study, preceding RVIs was one of the independent risk factors associated with severe pneumococcal pneumonia (a higher PSI score ≥91), suggesting a potential role of preceding RVIs on clinical severity. In previous studies, it was also reported that bacterial–viral co-infection in adults with CAP was associated with higher PSI risk than only bacterial infections.^10^–^13^ Hypoalbuminemia, azotemia, and host factors (i.e., male sex and increasing age) were also associated with the clinical severity of pneumococcal pneumonia, which are the known prognostic factors for mortality in patients with CAP.^6^–^9^

For both outpatients (13·6%) and hospitalized patients (86·4%) in our study, the in-hospital mortality rate of pneumococcal pneumonia was 8·4% compared to 11–20% for only hospitalized patients from other studies.^4^,^5^ There was no mortality among the 26 outpatients, which included 2 (7·7%) patients with severe pneumonia. The in-hospital mortality rate (4 [8·3%] versus 12 [8·4%], respectively; *P* = 1·000) and pneumococcal pneumonia-related mortality rate (4 [8·3%] versus 8 [5·6%], respectively; *P* = 0·501) were not significantly different between patients with preceding RVIs and those without preceding RVI.

In our study, the most frequent preceding RVI was influenza during the influenza season (November to April). Influenza virus infection has been demonstrated as an important predisposing factor for subsequent pneumococcal pneumonia. The hypothesized synergistic interactions between the two pathogens includes epithelial damage, changes in airway function, up-regulation of receptors, and changes to innate immune response.^19^–^21^ While the seasonal distribution of preceding RSV and HMPV infections overlapped during the winter and spring periods, the PIV infections were distributed from June to August. Interactions between pneumococcus and other respiratory viruses have also been suggested. RSV-induced impairments of macrophage or neutrophil function and cytokine signaling have increased the risk for pneumococcal infections in the murine model.^15^,^16^ HMPV or PIV and pneumococcus co-infections have also been demonstrated to act synergistically in the mouse model.^17^,^18^

During the study period, 25 (17·2%) of the 142 patients with other forms of bacterial pneumonia had co-infection with RVIs: *S. aureus* (*n* = 14), *H. influenzae* (*n* = 2), *P. aeruginosa* (*n* = 3) and *K. pneumoniae* (*n* = 6). In contrast to pneumococcal pneumonia, preceding RVIs were not significantly associated with severe pneumonia in patients with other form of bacterial pneumonia.

According to our study, screening and early detection of RVIs in patients with severe pneumococcal pneumonia might be warranted for appropriate antiviral therapy and the prevention of intra-hospital transmission. Prevention strategies, including vaccination against both seasonal influenza and pneumococcal diseases, have already been emphasized during the influenza season.

Our study has certain limitations. First, this was a single-center study. The limited number of case subjects who underwent laboratory tests for RVI might be associated with selection bias. The multiplex RT-PCR test for respiratory viruses has been introduced into clinical practice recently. Therefore, many clinicians have still a low level of test awareness and only the patients who had preceding or concurrent flu-like or cold symptoms were chosen for RVI testing. Secondly, the number of patients with RVIs other than influenza limited the ability to determine how different viral pathogens affect the clinical severity of pneumococcal pneumonia. Thirdly, this retrospective observational study included patients with pneumococcal pneumonia who received antibiotic therapy for ≥5 days based on the clinical diagnosis and outcome. Therefore, this study may not be representative of untreated patients of pneumococcal pneumonia. However, there were no patients who died within 5 days after admission among the 51 patients who had *S. pneumoniae* isolation from sputum cultures and received antibiotics therapy for <5 days. Fourthly, the incidence of pneumococcal bacteremia was low in our study; this might be partly due to the exposure of antibiotics before obtaining the first blood culture in 14 (7·3%) patients or the study design, which included only patients with pneumococcal pneumonia who had virological tests for RVI prior to isolating pneumococci from sputum cultures.

## Conclusion

In conclusion, preceding or concurrent RVIs might significantly influence the clinical severity in patients with pneumococcal pneumonia in addition to other factors, such as male sex, old age, hypoalbuminemia, and azotemia. Further studies are needed to understand the role of preceding or concurrent RVIs and to determine the clinical benefits of routine viral diagnostic tests for RVIs in patients with pneumococcal pneumonia.
